# Dynamic motor imagery mentally simulates uncommon real locomotion better than static motor imagery both in young adults and elderly

**DOI:** 10.1371/journal.pone.0218378

**Published:** 2019-06-26

**Authors:** Augusto Fusco, Luigi Iasevoli, Marco Iosa, Maria Chiara Gallotta, Luca Padua, Livia Tucci, Gabriella Antonucci, Carlo Baldari, Laura Guidetti

**Affiliations:** 1 IRCCS Fondazione Don Carlo Gnocchi, Milan, Italy; 2 Department of Movement, Human and Health Sciences, Section of Health Sciences, University of Rome "Foro Italico", Rome, Italy; 3 IRCCS Fondazione Santa Lucia, Rome, Italy; 4 Department of Geriatrics, Neurosciences and Orthopaedics, Sacred Heart Catholic University, Rome, Italy; 5 Fondazione Policlinico Universitario A. Gemelli IRCCS, Rome, Italy; 6 Department of Psychology, Sapienza University of Rome, Rome, Italy; 7 University eCampus, Rome, Italy; University of Canberra, AUSTRALIA

## Abstract

A new form of Motor Imagery (MI), called dynamic Motor Imagery (dMI) has recently been proposed. The dMI adds to conventional static Motor Imagery (sMI) the presence of simultaneous actual movements partially replicating those mentally represented. In a previous research conducted on young participants, dMI showed to be temporally closer than sMI in replicating the real performance for some specific locomotor conditions. In this study, we evaluated if there is any influence of the ageing on dMI. Thirty healthy participants were enrolled: 15 young adults (27.1±3.8 y.o.) and 15 older adults (65.9±9.6y.o.). The performance time and the number of steps needed to either walk to a target (placed at 10m from participants) or to imagine walking to it, were assessed. Parameters were measured for sMI, dMI and real locomotion (RL) in three different locomotor conditions: forward walking (FW), backward walking (BW), and lateral walking (LW). Temporal performances of sMI and dMI did not differ between RL in the FW, even if significantly different to each other (p = 0.0002). No significant differences were found for dMI with respect to RL for LW (p = 0.140) and BW (p = 0.438), while sMI was significantly lower than RL in LW (p<0.001). The p-value of main effect of age on participants’ temporal performances was p = 0.055. The interaction between age and other factors such as the type of locomotion (p = 0.358) or the motor condition (p = 0.614) or third level interaction (p = 0.349) were not statistically significant. Despite a slight slowdown in the performance of elderly compared to young participants, the temporal and spatial accuracy was better in dMI than sMI in both groups. Motor imagery processes may be strengthened by the feedback generated through dMI, and this effect appears to be unaffected by age.

## Introduction

Motor Imagery (MI) is a mental process during which the motor action is simulated without any overt motor output [1,2]. Research benefitted by considering imagery in the study of motor systems. In fact, imagery allows analysis of the processes of motor planning and motor execution both for simple and complex movements [3] and investigations of the mechanisms of motor control both in healthy participants and in patients with different diseases [4]. Techniques based on MI have been exploited as tools for improving motor performance in sport and as treatments in neurologic rehabilitation [5,6]. The imagery of movements shares many features with the actual executed actions, such as timing of actions, activations of neural substrates in several cortical and subcortical brain areas and neurophysiological autonomic responses [7–10]. In particular, MI consistently recruits a large fronto-parietal network in addition to subcortical and cerebellar regions [11]. Although the primary motor cortex is not consistently activated, the MI network includes several regions known as playing a role during actual motor execution [11]. Further, MI, as well as movement observation, may elicit physiological responses from the autonomic nervous system at the peripheral level; and in turn, these peripheral physiological responses may be analysed to provide objective evidences that MI is actually performed [12].

Recently, it has been proposed that the effectiveness of MI could be enhanced—through more accurately replicating the activation during actual motor performance—by including actual movements, performed simultaneously, with the mental representations of those same actions [13]. These actual movements are not the motor task itself, but simplified simulations of the movements involved in the real execution of the task. This type of “enriched” form of MI was previously presented by Gould and Damarjian [14] as a training based on “dynamic” imagery by replicating the physical movements made during the actual performance, even holding part of the equipment used in the performed sport in order to increase the vividness of imagery. Coupling movements with the same mental actions enhanced the confidence with an athletic gesture [15], probably influencing the neurophysiological processes of motor learning [16].

Hence, in this form of MI, called dynamic Motor Imagery (dMI), imagery is coupled with movements imitating some temporal or spatial features of the simultaneous mental representation of the action [13]. A previous study conducted on healthy young adults on locomotor imagery showed that the performance of dMI was different from that of classic motor imagery (static MI, sMI). However, dMI was closer to real performance than sMI for less common locomotor acts (such as lateral walking), but not for commonly experienced locomotion types (such as forward walking) [17].

It should be noted that many task-related conditions might influence MI performance. A body posture congruent or not congruent to that needed for the real execution of the movement during the imagery process can facilitate of inhibit MI, respectively [18,19]. The environment in which the movement is imagined can also influence the walking distance estimation [20], facilitating or not the mental imagery process [21], both for segmental movements of the upper limb [22] and for the whole-body movements [23,24]. Another possible hampering factor of the MI performances could be physical fatigue. In a recent work, Kanthack and colleagues have noted as the physical state could negatively influence the performances of athletes involved in accuracy tasks [25]. To take into account which elements can influence MI, the PETTLEP model has been proposed. This theoretical model can be easily applied in practice improving the planning and execution of imagery interventions [26, 27]. Originally proposed as a guideline for MI in athletes, this model can be extended to rehabilitation [28]. The PETTLEP model is based on seven components for making MI performance more similar to real ones and, in turn, for improving the real performance itself (see Table 1).

**Table 1:** The PETTLEP model.

Theoretical PETTLEP model			Application of PETTLEP model in our study
Component	sMI	dMI	
***Physical***	The participant should adopt the correct posture, wearing the same clothes and holding any implements that would be used during performance.	The participant should adopt the correct posture, wearing the same clothes and holding any implements that would be used during performance, moving them according to the performance, but remaining in place	The participant is standing on the starting line having the pathway in front of him/her for FW, behind for BW, laterally for LW. The stepping in place was performed moving the legs similarly to how they are moved during real performance.
***Environment***	The participant should complete the imagery in the same environment where the performance or task will take place (if impossible, videos, photographs, or a similar environment can be used as a substitute)	The participant should complete the imagery in the same environment where the performance or task will take place (if impossible, videos, photographs, or a similar environment can be used as a substitute)	The participant is on the starting line of the real pathway where the real performance will take place
***Task***	The task being imaged should be identical in nature to the task actually being performed, and this should be altered as the skill level of the participant improves.	The imagined task should be identical in nature to the task actually being performed, whereas the motor task is a simulation of the task to perform. The difference is that the simulated task is a simplified, in place, version of the real task, with the movements replicating some temporal or spatial features of the simultaneous mental representation of the action. Both imagery and simulated tasks should be altered as the skill level of the participant improves	The three tasks to imagine were identical to those to perform (FW, BW, LW). The task will be a stepping in place performed being in front of (FW), back to (BW) or laterally to (LW) the target. We did not take into account learning or changes in skill level during the experiment.
***Timing***	The imagery should be completed in “real time” and should take the same length of time to complete as physically performing the task.	The imagery should be completed in “real time” and should take the same length of time to complete as physically performing the task. Despite from a theoretical point of view the instruction is the same of sMI, a physical deficit may affect both the real performance as well as the simulated movement in dMI.	The time of the performance was one of the two outcomes we have evaluated to assess dMI and sMI versus RL. The other investigated parameter is the number of steps.
***Learning***	As the participant becomes proficient and autonomous at the task, the imagery should be updated in order to reflect this learning, and remain equivalent to the physical level of the participant.	As the participant becomes proficient and autonomous in the performance of the task, the imagery should be updated in order to reflect this learning and to remain equivalent to the physical level of the participant.	In our study, we did not plan a training that could imply a learning. On the contrary, we tried to avoid any learning that could have been a confounding factor in our study. For this reason, we recorded just one trial for each condition.
***Emotion***	Any emotions associated with performance should be incorporated into the imagery. This can be aided by the use of stimulus and response training.	Any emotion associated with the performance should be incorporated into the imagery. This can be aided by the use of stimulus and response training.	In our study, we did not give any particular instruction about emotions, and again we did not provide any feedback on the performance in order to avoid any learning effect.
***Perspective***	The imagery should usually be completed from an internal perspective (i.e., through the participant’s own eyes). This can be aided by the use of video. However, external imagery may be useful for some form-based tasks and personal preference should also be taken into account.	The imagery should usually be completed from an internal perspective (i.e., through the participant’s own eyes). This can be aided by the use of video. However, external imagery may be useful for some form-based tasks and also personal preferences should be taken into account.	In our study we used the first person perspective, being participants required to image the different walking patterns through their own eyes

The components of the PETTLEP model for static Motor Imagery (sMI), dynamic Motor Imagery (dMI), and how we applied this model in our study.

The PETTLEP model defines the components of MI. Some aspects of the personal attributes, such as age, can be lost by focusing on just the PETTLEP criteria alone. On the other hand, age or other personal factors may alter both imagery and real performances. The most common physiological effect of ageing seems to be a decline of MI performance, leading to longer response times [5,29–32]. Scientific literature showed that, during MI process, elderly need higher activations of primary motor area [33], supplementary motor area and prefrontal regions [34], together with compensatory activation of occipital lobes [35] than young participants. These compensatory processes highlighted in elderly were shown to be task-dependent [36]. For example, multisensory activation was found more prominent in elderly during standing than during walking, suggesting that the functional activations of the basic locomotor network (including prefrontal cortex, basal ganglia, brainstem, and cerebellar locomotor centres) are quite preserved in the elderly [37].

Currently, the possible relationship between ageing and dMI effectiveness has not been investigated. Together with the main effect of ageing, we also wanted to investigate if ageing has a task-specific effect on dMI, being the differences between dMI and sMI task-dependent. Hence, the main aim of this study was to characterise any differences between dynamic motor imagery with classic static motor imagery, versus the actual locomotion task of different walking conditions: in healthy young versus older participants. The hypothesis of this study was that dMI, coupling the mental representation of the locomotion with real imitating movements, may be more accurate than sMI in imagining the real performance also in older participants. This hypothesis is based on the potential beneficial effects of dMI related to the temporal advantage that this form of imagery offers. In fact, dMI shares some (simplified) biomechanical components with real locomotion (e.g. stepping place as opposed to real walking): eventual influence of age on physical performance could also influence the replicated movements, allowing participants to be more aware during imagination of their own physical status. In this scenario, dMI can be more helpful than sMI in adapted physical activity programs planned for the elderly to reduce the physical declined due to ageing or in rehabilitation for patients. The advantages of dMI with respect to real performance is that the simulated movements, being a simplified version of the real movements, can be performed with less fatigue by elderly or patients and also when motor deficits might limit the execution of the real movements. The advantages of dMI compared to sMI is that imagined timing may better take into account motor deficits thanks to the presence of sensorimotor feedback quite similar to those occurring during the real task.

Because the process related to the formation of mental locomotor imagery seems to be dependent on the type of locomotor act [17,38], we have investigated dMI and sMI in different types of locomotion. We tested locomotion in the forward walking (FW, which is the most commonly performed automatic and cyclical action for the natural human locomotion), backward walking (BW, which is biomechanically similar to FW, but rarely performed) and lateral walking (LW, which is less common and biomechanically different with respect to FW). In lateral walking (LW) condition, participants were asked to advance laterally, so that one leg (self-chosen by participants) was always in front of the other (in the direction of the movement). Backward walking (BW) was chosen as unusual walking having kinematics similar to that of the FW [39], even if the BW is generally slower with respect to FW [40]. The speed required to perform a given task has been found to influence the duration of MI [41].

Previous studies on the classic form of MI showed a temporal similarity among different ranges of age only for movements coherent with usual physical practice [42] and in the short/middle distances of locomotion (considered up to 19 meters) [43,44]. On the contrary, it resulted in age-dependent effects for other types of movements as complex actions (for example, arm pointing task) [43] or constrained ones [29,44]. In this scenario, it is conceivable that age may affect LW, partially BW, and not FW.

## Material and methods

### Protocol

We designed the study according to a protocol already used to assess the spatial and temporal parameters of motor imagery of different locomotor tasks in previous studies [17,20,38]. Naïve individuals were asked to imagine walking in different conditions towards a visualized target without any overt associated movement maintaining a posture congruent with the relevant actual locomotion (static Motor Imagery, sMI). Then, they were asked to imagine the same different types of walking accompanied by a simultaneous stepping in place performed with a posture and a manner both coherent with the actual execution of that movement (dynamic Motor Imagery, dMI). The given locomotor conditions were: forward walking (FW), backward walking (BW) and lateral walking (LW).

In light of the evidence of improved effectiveness of internal visual imagery, versus external visual imagery [45], a researcher instructed all the participants on how to perform the task from a first-person visual perspective. Hence, they were required to image the different walking patterns through their own eyes, moving themselves into the environment (indoor setting) up to the target line.

Participants stood on a line marked on the floor in front of another line taped on the ground at a distance of 10 m, unknown to the participants, fixing the target. Because body posture and environment have been shown to influence MI performances [18,20], the participants always performed the tasks in indoor setting, standing: both when imagining or actually doing the task. The target was positioned in front of them in FW, behind them in BW, and laterally to them in LW; so that the starting position was congruent to the type of required locomotion. All the participants had a normal or corrected-to-normal vision. Before starting the trial, they were asked to verbally judge the distance expressing it in meters. Participants completed the real or imagined walking task at self-paced speed, without any further specific instructions concerning: movement duration; velocity; nor familiarization sessions to the experimental procedure. The FW was performed in absence of any specific instructions. During BW condition, participants were placed with their back to the target. At the beginning of the trial, looking the target over their own shoulder was required, leaving participants free to choose the preferred side for the head rotation. Then, they were left free to continue or not to look at the target. In the LW, participants chose the laterality according to the preferential direction in which starting the task. Subsequently, researchers furnished details about how performing the LW in order to preserve the same conditions for all participants. Participants were requested to imagine lateral walking keeping one foot still while oscillating the other one in the frontal plane, simulating the lateral body displacement occurring during LW. Participants were also asked not to cross the two legs. Real locomotion (RL) was performed after imagery tasks. Hence, in our protocol, the sequence of locomotor conditions was fixed (sMI-dMI-RL), and not randomized, in order to avoid the possible influence of RL or dMI on the sMI performance. The opposite (sMI on dMI) was less probable due to the lack of sensorimotor feedback in sMI. Further, no feedback was provided to participants about their performances to avoid a possible learning driven by a formal feedback. In order to counterbalance the experiment, only the different locomotion was randomized. To avoid any learning effect, just one trial for each experimental condition was tested.

The duration of imagined movements was recorded by the enrolled participants with a sport chronograph digital timer stopwatch (JUNSD), with a time resolution of 0.1 s. Participants were asked to press the button of the stopwatch when they mentally started the task and when they considered they had arrived at the target, according to the mental chronometry [46]. The temporal and spatial performances (time and number of steps needed to perform the different locomotor conditions) were recorded by means of an accelerometer located with a belt at the third lumbar spinous process, in correspondence of the body centre of mass, during dMI and real locomotion tasks. Accelerometry is a quantitative technique suitable for objectively estimating spatiotemporal parameters of locomotion, reliable and sensitive in physiological as well as in pathological walking [47,48]. Finally, individuals were asked to really perform the task (real locomotion, RL), walking actively and with full vision towards the target. Again, the time of execution and the number of performed steps were recorded by the accelerometric signals. As double-check, the number of steps was also validated by an experimenter making a visual count of them during the execution of the trial. A software was developed in MATLAB (The MathWorks, Inc.) and used to identify the minima of cranio-caudal acceleration during the performance: each peak corresponding to a foot strike and hence to a single step [20].

The study was conducted in accordance with the Declaration of Helsinki about experiments on human participants and it was approved by the independent local ethical committee of the Hospital (IRCCS Fondazione Santa Lucia). Informed consent was obtained from the participants before starting the trial.

### Participants

Thirty healthy volunteers were enrolled for two groups: young adults (15 participants, 6 males and 9 females; mean age: 27.1±3.8 years old) and older adults (15 participants, 8 males and 7 females; mean age: 65.9±9.6 years old). In accordance with the study design, age resulted significantly different between groups (p<0.0001, t-test). None of the participants reported a history of neurological or orthopaedic disease influencing the locomotion, nor did any report taking psychoactive or vasoactive medication. None had cognitive impairment, as assessed by Mini-Mental State Examination. All the participants performed the experiment wearing a comfortable, usually used, pair of shoes.

### Statistical analyses

Sample size was chosen according to previous studies [17,20]. Means and standard deviations were computed for all the investigated parameters. Temporal and spatial performance of the different imagery conditions (sMI and dMI) were also estimated in terms of absolute and percent values with respect to the real locomotion in three different walking conditions (comparing each motor imagery performance with the relevant actual locomotor task). Repeated measure analysis of variance (RM-ANOVA) was performed using the imagery condition (sMI, dMI or RL) as within participant factor and the locomotor condition (forward walking, backward walking and lateral walking) and age as between group factors. Values of partial eta-squared were reported together with F- and p-values for assessing the effect size (ES) of each factor. The alpha level defining the statistically significant threshold was set at 0.05. However, for post-hoc analysis, performed when needed, this threshold was corrected in accordance to Bonferroni, taking into account the number of possible comparisons (for time: 9 comparisons were possible and hence the alpha level was reduced at 0.05/9 = 0.0056, whereas for the number of steps: 6 comparisons were possible and alpha level was reduced at 0.05/6 = 0.0083). Sample size was fixed at 15 participants for each group according to preliminary data [35], in which 12 elderly participants were included.

Moreover, the Imagery Performance Index (IPI) was also computed as the absolute and relative difference between the performance during motor imagery and the relevant performance recorded during actual execution of the same locomotor task expressed in percentages of actual performance value. A mixed ANOVA was performed with age-related group as factor between participants (young adults vs. elderly) and with two factors within participant: type of motor imagery (sMI vs. dMI) and locomotor condition (FW, BW and LW). The correlation between the perceived distance and the percentage variation of time evaluated between imagery and real performances was tested using Pearson’s correlation coefficient (R).

Parametric statistics were chosen because of continuous normally distributed data. For all experiments, all measures, in all conditions, the entire data-set was analysed, without excluding any data, also because we did not observe any data classifiable as outliers.

## Results

The performances of participants resulted timely different with respect to locomotor condition and imagery condition, as shown by the results of RM-ANOVA reported in Table 2.

**Table 2:** Results of statistical analyses on time performance.

Factor		F	P	ES
**Main factors**	**Imagery condition**	F(2,56) = 13.892	**p<0.001**	0.322
	**Locomotor condition**	F(2,56) = 32.426	**p<0.001**	0.537
	**Age**	F(1,28) = 4.021	p = 0.055	0.126
**Age interaction with**	**Imagery condition**	F(2,56) = 1.048	p = 0.358	0.036
	**Locomotor condition**	F(2,56) = 0.492	p = 0.614	0.017
**Interaction**	Imagery Condition * Locomotor Condition	F(4,112) = 6.175	**p<0.001**	0.181
**III level interaction**	Imagery condition* Locomotor condition* Age	F(4,112) = 1.124	p = 0.349	0.039

Results of Mixed Repeated Measure Analysis of Variance: F (degrees of freedom), p-values and ES (Effect Size, partial eta squared). In bold, it was reported values statistically significant.

Significant effects of imaginary condition and locomotor condition were found, whereas there was not a significant effect of age. Further, neither significant interactions of age with motor imagery condition nor with locomotor condition were found (p>0.05 for all the interactions involving age factor). The only significant interaction was between locomotor and imagery conditions. These results showed that the differences between dynamic and static motor imagery performances resulted statistically significant only in specific locomotor conditions. This result was observed in both groups, regardless of their age.

Post-hoc comparisons showed that significant differences were mainly related to the differences between imagination and actual locomotion observed in LW. Conversely, no specific differences were found for the other two walking conditions (FW, BW). Moreover, post-hoc analysis showed that the sMI and dMI performances resulted different for FW (p = 0.0002; subgroups: young adults: p = 0.0051; older adults: p = 0.0037) despite both resulted not significantly different from RL performances.

Conversely, the estimation of time needed to reach the target during sMI resulted significantly lower than during RL (p<0.001) and dMI performances (p<0.001). Conversely, RL and dMI performances were not significantly different for lateral walking (p = 0.140) as well as for forward (p = 0.099) and backward (p = 0.438) walking.

**Fig 1:**
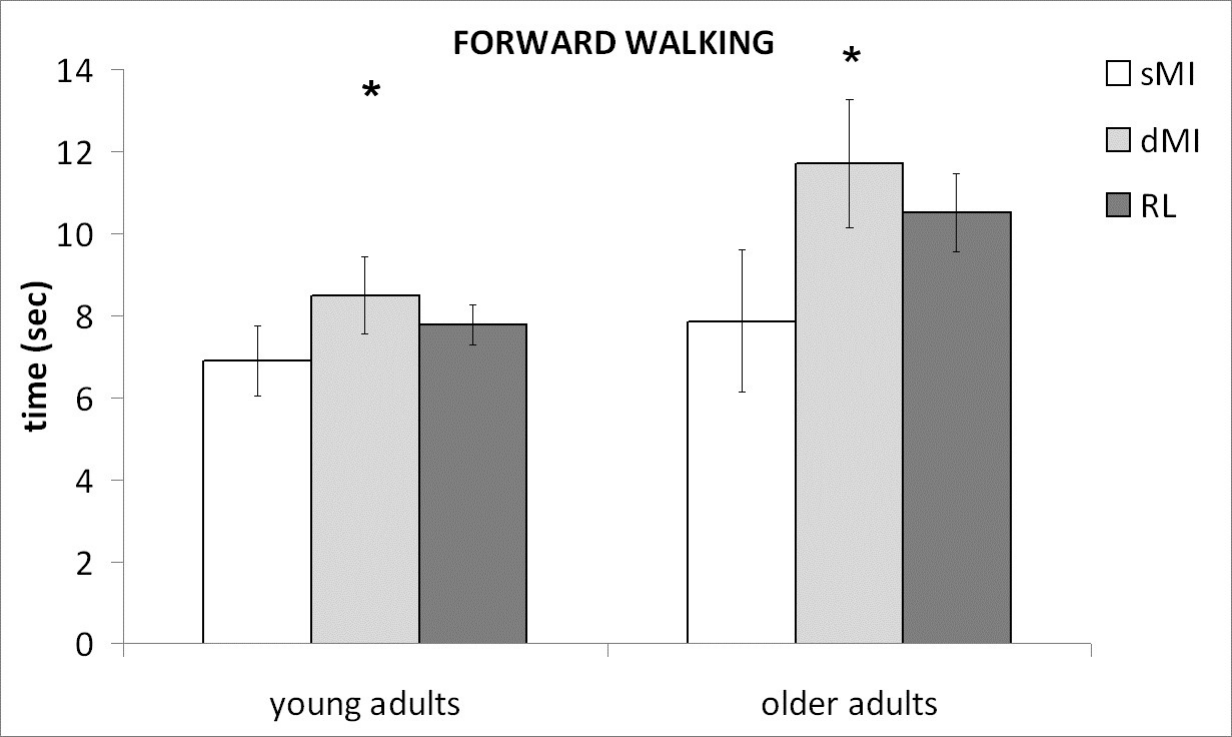
Temporal performances in forward walking. Mean and standard deviations in static and dynamic Motor Imagery (sMI, dMI) and actual physical execution (Real Locomotion, RL) in forward walking. Stars indicate the significant differences between sMI and dMI.

**Fig 2:**
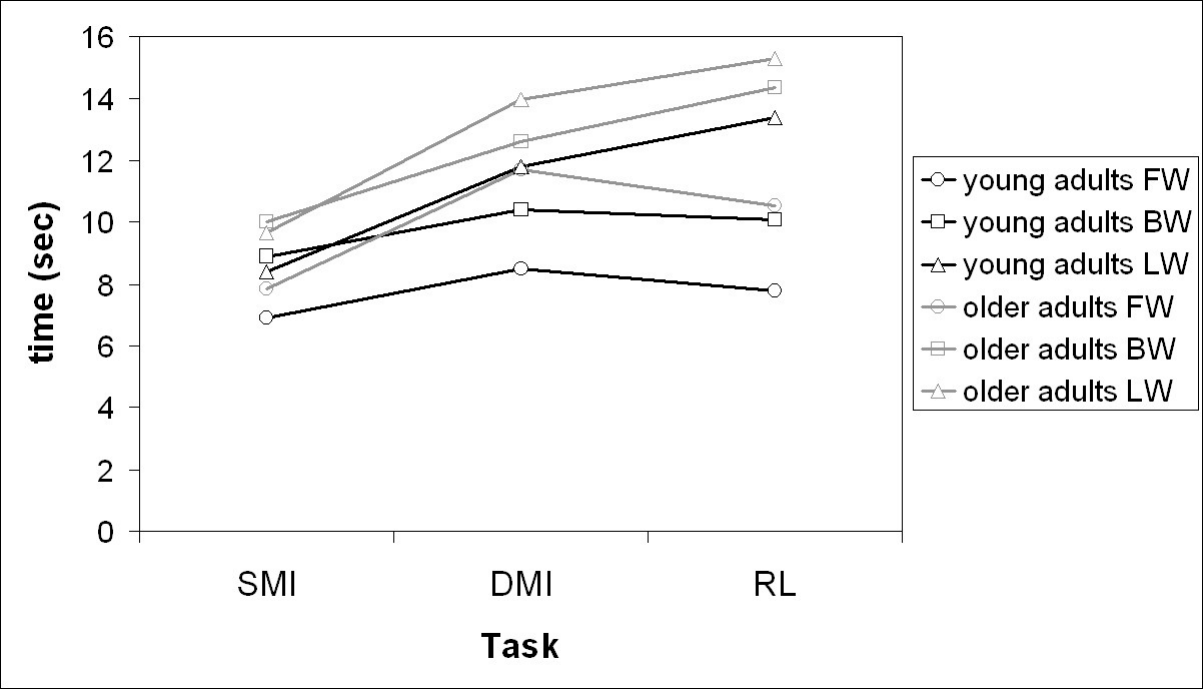
Temporal performances for the different tasks of young and older adults. Mean of time reported in seconds for every tasks (standard deviations and significant differences have been not reported for the sake of clarity of the figure).

Fig 1 shows the time spent for executing the imagery tasks (dMI and sMI) and the actual locomotion for FW in the different groups, while the temporal performances of the tasks for the different locomotor conditions are summarized in Fig 2.

Globally, we observed a non-significant trend in which the young group performed faster than elderly all the different tasks. Conversely, the dMI performance was temporally more similar to that of RL than SMI (Table 3). This result was found in both groups, without any specific effect of age (p = 0.358).

**Table 3:** Results of temporal performance of the enrolled participants. Mean and standard deviation (SD) are reported in the first three columns (in seconds), difference (in seconds) and percent variation (%) in the last two ones.

	**YOUNG GROUP**				
	*sMI (SD)*	*dMI (SD)*	*RL (SD)*	*Absolute difference and percent variation* *sMI-RL*	*Absolute difference and percent variation dMI-RL*
**FW**	6.9 (2.1)	8.5 (2.3)	7.8 (1.2)	- 0.8; -10.0%	+ 0.7; 10.6%
**BW**	8.9 (3.2)	10.4 (4.1)	10.1 (1.9)	- 1.2; -10.9%	+ 0.3; 5.0%
**LW**	8.4 (3.1)	11.8 (3.8)	13.4 (3.8)	- 5.0; -32.2%	- 1.6; -5.5%
	**ELDERLY GROUP**				
	*sMI (SD)*	*dMI (SD)*	*RL (SD)*	*Absolute difference and percent variation* *sMI-RL*	*Absolute difference and percent variation dMI-RL*
**FW**	7.9 (4.2)	11.7 (3.8)	10.5 (2.3)	- 2.6; -23.8%	+ 1.2; 13.6%
**BW**	10.0 (6.5)	12.6 (4.7)	14.4 (5.9)	- 4.4; -26.7%	- 1.8; -7.3%
**LW**	9.6 (5.8)	14.0 (6.5)	15.3 (4.3)	- 5.7; -38.1%	- 1.3; -8.5%

The mixed-ANOVA performed on the Imagery Perfomance Index (IPI) computed on the absolute difference between motor imagery and actual performances did not show any significant effect. Conversely, the IPI computed on the raw difference (not absolute values) was found significantly affected by the type of motor imagery (F(1,28) = 33.325, p<0.001) and by the type of locomotion (F(2,56) = 7.394, p = 0.001). These two factors seemed to affect IPI independently of each other, as shown by the absence of any significant interaction between them on results (F(2,56) = 2.155, p = 0.125). Age did not play a significant role on IPI, neither as main factor (F1,28 = 0.683, p = 0.416) nor interacting with other factors, (interactions of age with locomotor conditions: F(2,56) = 0.602, p = 0.551, with type of motor imagery: F(1,28) = 0.790, p = 0.382, and with both of them F(2,56) = 0.839, p = 0.438).

**Fig 3:**
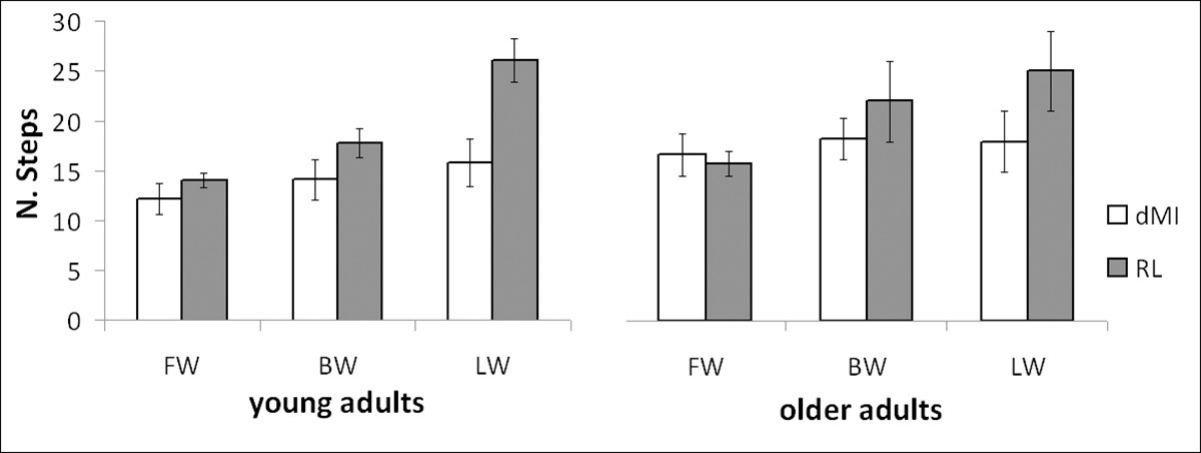
Step performances for the different tasks of young and older adults. Number of performed steps in the different locomotor conditions for both groups for imagery tasks and real physical practice.

The number of steps was analysed between dMI and the relevant actual performances (Fig 3). Analogously, the effect of task execution (dMI vs. RL) and locomotor conditions resulted statistically significant (F(1,28) = 17.192, p<0.001; F(2,56) = 36.698, p<0.001; respectively), as well as their interaction (F(2,56) = 21.819, p<0.001). Again, age did not affect the results (F(1,28) = 2.300, p = 0.141), despite a quite (not significant) effect of the interaction age per condition on the number of performed steps (F(2,56) = 2.999, p = 0.058). Post-hoc analysis showed a significant difference between dMI and RL performances only for lateral walking (p<0.001), just partial on BW (p = 0.096, not significant for the Bonferroni correction) and absent for FW (p = 0.594).

Finally, the perceived distance could have been a possible confounding factor in relation to the age of participants, but it was not significantly different between young adults and elderly (7.03±2.00m vs. 7.77±2.95m, p = 0.432). When we tested the possible correlation between this perceived distance and the percentage variation of performances reported in Table 3, significant correlations were found only for EG and not for YG. In particular, perceived distance resulted significantly correlated with sMI performance in LW (R = 0.525, p = 0.045) and with dMI performance in FW (R = 0.772, p = 0.001).

## Discussion

The aim of this study was to evaluate dynamic motor imagery (dMI) in two groups of healthy participants of different age, comparing it with the classic form of motor imagery (static Motor Imagery, sMI) in different types of locomotion. Despite a slight non-significant trend related to a slowed-down performance in the elderly group, the better ability of dMI with respect to sMI to temporally simulate actual execution of uncommon types of locomotion resulted similar in young and older participants. The longer temporal performances of older adults with respect to those of young adults occurred for the sMI, for the dMI as well as for the actual locomotion (RL). The longer time for older adults may reflect that they actually moved slower, and their imagery accurately reflects that. Zwergal and colleagues [37] observed that locomotor imagery is well conserved in elderly. The age of our elderly was lower than that of elderly groups enrolled in other studies in which age was found significantly affecting MI [29,32].

According to previous results [17], also in our study we found a significant effect of imagery condition (main effect of dMI vs. sMI) and a significant interaction between imagery condition and locomotor condition. In general, the time spent by participants in sMI resulted significantly lower than that needed during RL. Conversely, the temporal performance recorded during dMI, with respect to RL, resulted significantly shorter in LW, and not significantly different in FW and BW. Forward walking resulted the only locomotor condition without any significant difference between sMI and RL performances, as well as dMI and RL performances (despite a significant difference between sMI and dMI). This could be due to the fact that forward walking is a well-learned, reliable and standardized motor skill [49].

A previous study showed that temporal performances during dMI were more similar to real performances than sMI for light running and lateral walking [17]. Differently from that study, we evaluated older adults and, for this reason, we have excluded light running condition. For the other locomotor conditions (BW and LW), that are heavily practised (being less common than FW), our results confirmed a better time accuracy of dMI. Our results support the idea that dynamic motor imagery benefit by the mental perception of timing given by the stepping in place that provides useful feedback in order to obtain times similar to actual performance both in young and older adults [17,50]. Another potential factor affecting performance is the perceived distance. Although it did not differ between the two groups, the elderly showed a significant correlation between the perceived distance and the percentage variation of temporal performances: the longer the perceived distance was, the longer the imagination timing was. It implied that elderly perceiving a longer distance showed a reduction of erroneous anticipation in LW during sMI, but also an increased erroneous delay in FW during dMI. These results highlight the importance to take into account the perceived distance as a factor playing a role in the time estimation during both sMI and dMI for elderly more than for young participants, despite not directly deferring between the two groups.

The duration of sMI of both groups tended to be constant quite independently by locomotor condition (ranging between 7 and 10s), while both dMI (ranging between 8.5 and 14s) and RL (ranging between 8 and 15s) were much more variable for both groups. In sport scientific literature, whole-body complex tasks have been reported as imagined temporal performances quicker than those of the actual performance [51–53], possibly for the complexity of the requested motor skill and the expertise of the involved participants. Furthermore, novices were shown to have faster imagery timings than experts [53]. These findings seem to be in accordance with the poor sMI performance in LW that is an unusual type of locomotion. Furthermore, our participants showed similar temporal performances among different imagined locomotor conditions in sMI. On the contrary, the temporal performance was more variable among locomotor conditions during dMI as well as during real locomotion. We found non-significant differences between young subjects and our elderly group during real performances. It could be due to the fact that our elderly group was, on average, 66 years old. Different results could be found for people older than 80 years for their physical decline due to aging [5,43] and for longer path [44].

Spatial and temporal parameters were not significantly influenced by execution condition both for forward and backward walking. The kinematics of BW has previously been reported to be similar to that of FW [39]. In BW, the participant’s speed is generally decreased compared to FW, as confirmed in our study, while the characteristics of angular displacement in all joints in BW are similar, but time-reversed, to those of FW [40]. The only locomotor condition that resulted greatly influencing the motor imagery was the lateral walking. This is another unusual locomotor condition, but in this case, the performances can be affected by three possible confounding factors. Because participants were asked to laterally walk without crossing the standing foot with the swinging foot (ahead or behind the participant), this constraint might have affected the data, but it was fundamental to be sure that imagined and actual movement really executed were similar, according to the PETTLEP model presented in this study. In literature, it has been reported that every form of constraints or incongruent postures, such as imagining a walk being sit [18] or walking on narrow [29], can negatively influence the MI. Another possible reason is the different kinematics related to the different muscles involved in lateral walking, with hip ab-adductors muscles more involved than hip flexo-extensors that are usually involved in forward walking. Finally, the third possible explanation can be related to the locomotor body schema [54], i.e. an internal model of walking helpful for estimating the time and the number of steps needed to reach a target placed at a given distance. Hence, motor imagery can be considered as a task-dependent process, i.e. depending on the type of movement, probably intertwined with an internal model of FW [38,55].

### Study limitation

Our study had some limitations to take into account. The first one was the use of only one target-distance. Participants might have simply recalled to memory the time estimated in the previous imagery condition or locomotor condition. The sequence of execution conditions (sMI, dMI, RL) was chosen because hypothesized as the one less influencing participants. On the other hand, if participants simply used the same time of the first condition, no different results would have been obtained. However, a potential bias of using just one distance could have influenced the results. However, one distance and just one trial recorded for each experimental condition allowed us to avoid a learning effect that, despite learning is a fundamental component of PETTLEP model, it was out of the scope of our study.

Another limit is the sample size of this study. Probably a wider sample allowed highlighting also a significant main effect of age. Nevertheless, we were more interested in interactions between age and other factors, which resulted far from any statistically significant result, and hence probably not affected by the sample size.

Another limit of our study is the absence of a measure of imagery ability assessed by one of the scales proposed in literature, such as the MIQ-3 [56].

Further studies should be include more target distances, presented in a randomized order, to a wide sample of participants. A wider sample may increase the power of statistical tests, despite in this study, the number of participants, chosen according to previous studies [17,20], resulted sufficient to highlight statistically significant results. Also the imagery condition should be randomized, and the imagery ability should be assessed with a validated scale.

### Perspectives

Despite its limits, our study is particularly interesting at the light of the clinical perspectives. In fact, the use of MI has been demonstrated to be a valuable treatment for rehabilitation in most of the neurological disorders [57]. Studies have shown as cognitive decline may result in the deterioration of gait patterns, affecting also the motor imagery processes [18,58]. Further investigations should be focused on the locomotor imagery in pathological conditions. In a recent study, Iosa and colleagues showed as dMI and RL performances were more consistent in adults than in children, and greatly altered in children with cerebral palsy [55]. Future researches should elucidate which factors can influence the effectiveness of possible treatment developed in the MI process. One of the possible hampering factors is physical fatigue, which could also influence imagery accuracy, altering the temporal organization of motor imagery [25]. Consistently with this assumption, also constrained or unusual movements (as LW in our experiment) can impair the imagery accuracy and effectiveness. In the pathological condition, it is necessary to assess carefully the state of cognitive decline of the elderly into imagery formation and perception. In our experiment, we did not analyze the motor imagery ability by means of a questionnaire, and that was another limitation of our study. This factor will have to be deeply analyzed in further studies with a wider sample size to understand how imagery features of patients may influence different outcomes. It has been shown as recovery can be more difficult in some type of individuals: the use of correct questionnaires could be helpful to verify the possible patient adequate to the treatment.

## Conclusions

We have examined whether age affected the performance related to dynamic and static motor imagery in a manner depending on the type of locomotor act. Our results showed as the performances of dynamic motor imagery remained closer to real performance than classical static motor imagery especially in uncommon locomotor tasks also for the elderly. Furthermore, this is the first study defining the application of the PETTLEP model to dynamic motor imagery, providing interesting perspectives in neurorehabilitation and adapted physical activity programs contrasting ageing decline in elderly.

## Supporting information

S1 File Dataset. In the file, it is reported the dataset of the experiment for each participant.
(XLSX)

## References

[1] Jeannerod M. The representing brain: neural correlates of motor intention and imagery. Behav Brain Sci. 1994;17(2):187–245. 10.1017/S0140525X00034026

[2] Decety J, Grèzes J. Neural mechanisms subserving the perception of human actions. Trends Cogn Sci. 1999;3(5):172–178. 10.1016/S1364-6613(99)01312-1 10322473

[3] Caeyenberghs K, Wilson PH, van Roon D, Swinnen SP, Smits-Engelsman CM. Increasing convergence between imagined and executed movements across development: evidence for the emergence of movement representations. Dev Sci. 2009;12(3):474–83. 10.1111/j.1467-7687.2008.00803.x 19371372

[4] de Lange FP, Roelofs K, Toni I. Motor imagery: A window into the mechanisms and alterations of the motor system. Cortex. 2008;44(5):494–506. 10.1016/j.cortex.2007.09.002 18387583

[5] Malouin F, Richards CL. Mental practice for relearning locomotion skills. Phys Ther. 2010;90(2):240–51. 10.2522/ptj.20090029 20022993

[6] Schack T, Essig K, Frank C, Koester D. Mental representation and motor imagery training. Front Hum Neurosci. 2014;8:328 10.3389/fnhum.2014.00328 24904368 PMC4033090

[7] Guillot A, Collet C. Duration of mentally simulated movement: a review. J Mot Behav. 2005;37(1):10–20. 10.3200/JMBR.37.1.10-20 15642689

[8] Guillot A, Collet C, Nguyen VA, Malouin F, Richards C, Doyon J. Functional neuroanatomical networks associated with expertise in motor imagery ability. Neuroimage. 2008;41(4):1471–83. 10.1016/j.neuroimage.2008.03.042 18479943

[9] Munzert J, Lorey B, Zentgraf K. Cognitive motor processes: the role of motor imagery in the study of motor representations. Brain Res Rev. 2009;60(2):306–26. 10.1016/j.brainresrev.2008.12.024 19167426

[10] Macuga KL, Frey SH. Neural representations involved in observed, imagined, and imitated actions are dissociable and hierarchically organized. Neuroimage. 2012;59(3):2798–807. 10.1016/j.neuroimage.2011.09.083 22005592 PMC3254825

[11] Hétu S, Grégoire M, Saimpont A, Coll MP, Eugène F, Michon PE et al The neural network of motor imagery: an ALE meta-analysis. Neurosci Biobehav Rev. 2013;37(5):930–49. 10.1016/j.neubiorev.2013.03.017 23583615

[12] Collet C, Di Rienzo F, El Hoyek N, Guillot A. Autonomic nervous system correlates in movement observation and motor imagery. Front Hum Neurosci. 2013 7 30;7:415 10.3389/fnhum.2013.00415 23908623 PMC3726866

[13] Guillot A, Moschberger K, Collet C. Coupling movement with imagery as a new perspective for motor imagery practice. Behav Brain Funct. 2013;9:8 10.1186/1744-9081-9-8 23425312 PMC3599464

[14] Gould D, Damarjian N. Imagery training for peak performance In Van Raalte JL, Brewer BW, Editors. Exploring sport and exercise psychology. Washington, DC, US: American Psychological Association; 1996 pp. 25–50. 10.1037/10186-002

[15] Callow N, Roberts R, Fawkes JZ. Effects of dynamic and static imagery on vividness of imagery, skiing performance, and confidence. J Im Res Sport Phys Act. 2006;1:1–13. 10.2202/1932-0191.1001

[16] Debarnot U, Sperduti M, Di Rienzo F, Guillot A. Experts bodies, experts minds: How physical and mental training shape the brain. Front Hum Neurosci. 2014;8:280 10.3389/fnhum.2014.00280 24847236 PMC4019873

[17] Fusco A, Iosa M, Gallotta MC, Paolucci S, Baldari C, Guidetti L. Different performances in static and dynamic imagery and real locomotion. An exploratory trial. Front Hum Neurosci. 2014 10 2;8:760 10.3389/fnhum.2014.00760 25324758 PMC4183108

[18] Saimpont A, Malouin F, Tousignant B, Jackson PL. The influence of body configuration on motor imagery of walking in younger and older adults. Neuroscience. 2012;222:49–57. 10.1016/j.neuroscience.2012.06.066 22796073

[19] Aglioti SM, Cesari P, Romani M, Urgesi C. Action anticipation and motor resonance in elite basketball players. Nat Neurosci. 2008;11(9):1109–16. 10.1038/nn.2182 19160510

[20] Iosa M, Fusco A, Morone G, Paolucci S. Walking there: environmental influence on walking-distance estimation. Behav Brain Res. 2012;226(1):124–32. 10.1016/j.bbr.2011.09.007 21925542

[21] Guillot A, Collet C, Dittmar A. Influence of environmental context on motor imagery quality. Biol Sport. 2005 22:215–26.

[22] Papaxanthis C, Schieppati M, Gentili R, Pozzo T. Imagined and actual arm movements have similar durations when performed under different conditions of direction and mass. Exp Brain Res. 2002;143(4):447–52. 10.1007/s00221-002-1012-1 11914790

[23] Munzert J. Temporal accuracy of mentally simulated transport movements. Percept Mot Skills. 2002;94(1):307–18. 10.2466/pms.2002.94.1.307 11883579

[24] Papaxanthis C, Pozzo T, Kasprinski R, Berthoz A. Comparison of actual and imagined execution of whole-body movements after a long exposure to microgravity. Neurosci Lett. 2003;339(1):41–4. 10.1016/S0304-3940(02)01472-6 12618296

[25] Kanthack TF, Guillot A, Altimari LR, Nunez Nagy S, Collet C, Di Rienzo F. Selective efficacy of static and dynamic imagery in different states of physical fatigue. PLoS ONE. 2016 3 1;11(3):e0149654 10.1371/journal.pone.0149654 26930279 PMC4773141

[26] Holmes PS, Collins DJ. The PETTLEP approach to motor imagery: a functional equivalence model for sport psychologists. J Applied Sport Psych. 2001;13(1):60–83. 10.1080/10413200109339004

[27] Wakefield C, Smith D. Perfecting Practice: applying the PETTLEP Model of motor imagery. J Sport Psych in Action. 2012;3(1):1–11. 10.1080/21520704.2011.639853

[28] Harris JE, Hebert A. Utilization of motor imagery in upper limb rehabilitation: a systematic scoping review. Clin Rehabil. 2015 11;29(11):1092–107. 10.1177/0269215514566248 25604911

[29] Personnier P, Kubicki A, Laroche D, Papaxanthis C. Temporal features of imagined locomotion in normal aging. Neurosci Lett. 2010;476(3):146–9. 10.1016/j.neulet.2010.04.017 20399251

[30] Personnier P, Paizis C, Ballay Y, Papaxanthis C. Mentally represented motor actions in normal aging II. The influence of the gravito-inertial context on the duration of overt and covert arm movements. Behav Brain Res. 2008;186(2):273–83. 10.1016/j.bbr.2007.08.018 17913253

[31] Skoura X, Personnier P, Vinter A, Pozzo T, Papaxanthis C. Decline in motor prediction in elderly subjects: right versus left arm differences in mentally simulated motor actions. Cortex. 2008 10;44(9):1271–8. 10.1016/j.cortex.2007.07.008 18761141

[32] Saimpont A, Pozzo T, Papaxanthis C. Aging affects the mental rotation of left and right hands. PLoS ONE. 2009;4(8):e6714 10.1371/journal.pone.0006714 19707585 PMC2726952

[33] Sharma N, Baron JC. Effects of healthy ageing on activation pattern within the primary motor cortex during movement and motor imagery: an fMRI study. PLoS ONE. 2014;9(6):e88443 10.1371/journal.pone.0088443 24887402 PMC4041563

[34] Allali G, van der Meulen M, Beauchet O, Rieger SW, Vuilleumier P, Assal F. The neural basis of age-related changes in motor imagery of gait: an fMRI study. J Gerontol A Biol Sci Med Sci. 2014 11;69(11):1389–98. 10.1093/gerona/glt207 24368777

[35] Zapparoli L, Saetta G, De Santis C, Gandola M, Zerbi A, Banfi G, et al When I am (almost) 64: The effect of normal ageing on implicit motor imagery in young elderlies. Behav Brain Res. 2016 4 15;303:137–51. 10.1016/j.bbr.2016.01.058 26851363

[36] Berlingeri M, Bottini G, Danelli L, Ferri F, Traficante D, Sacheli L, et al With time on our side? Task-dependent compensatory processes in graceful aging. Exp Brain Res. 2010;205(3):307–24. 10.1007/s00221-010-2363-7 20680252

[37] Zwergal A, Linn J, Xiong G, Brandt T, Strupp M, Jahn K. Aging of human supraspinal locomotor and postural control in fMRI. Neurobiol Aging. 2012;33(6):1073–84. 10.1016/j.neurobiolaging.2010.09.022 21051105

[38] Fusco A, Gallotta MC, Iosa M, Morone G, Iasevoli L, Trifoglio D, et al The dynamic motor imagery of locomotion is task-dependent in patients with stroke. Restor Neurol Neurosci. 2016;34(2):247–56. 10.3233/RNN-150573 26889966

[39] Viviani P, Figliozzi F, Campione GC, Lacquaniti F. Detecting temporal reversals in human locomotion. Exp Brain Res. 2011;214(1):93–103. 10.1007/s00221-011-2809-6 21814834

[40] Lee M, Kim J, Son J, Kim Y. Kinematic and kinetic analysis during forward and backward walking. Gait Posture. 2013;38(4):674–8. 10.1016/j.gaitpost.2013.02.014 23541766

[41] Louis M, Guillot A, Maton S, Doyon J, Collet C. Effect of imagined movement speed on subsequent motor performance. J Mot Behav. 2008 3;40(2):117–32. 10.3200/JMBR.40.2.117-132 18400678

[42] Saimpont A, Malouin F, Tousignant B, Jackson PL. Motor imagery and aging. J Mot Behav. 2013;45(1):21–8. 10.1080/00222895.2012.740098 23394362

[43] Skoura X, Papaxanthis C, Vinter A, Pozzo T. Mentally represented motor actions in normal aging. I. Age effects on the temporal features of overt and covert execution of actions. Behav Brain Res. 2005;165(2):229–39. 10.1016/j.bbr.2005.07.023 16165229

[44] Schott N, Munzert J. Temporal accuracy of motor imagery in older women. Int J Sport Psych. 2007;38(3):304–320.

[45] Callow N, Roberts R, Hardy L, Jiang D, Edwards MG. Performance improvements from imagery: evidence that internal visual imagery is superior to external visual imagery for slalom performance. Front Hum Neurosci. 2013;7:697 10.3389/fnhum.2013.00697 24155710 PMC3803114

[46] Schott N. Age-related differences in motor imagery: working memory as a mediator. Exp Aging Res. 2012;38(5):559–83. 10.1080/0361073X.2012.726045 23092223

[47] Iosa M, Morelli D, Marro T, Paolucci S, Fusco A. Ability and stability of running and walking in children with cerebral palsy. Neuropediatrics. 2013;44(3):147–54. 10.1055/s-0033-1336016 23487325

[48] Iosa M, Picerno P, Paolucci S, Morone G. Wearable inertial sensors for human movement analysis. Expert Rev Med Devices. 2016;13(7):641–59. 10.1080/17434440.2016.1198694 27309490

[49] Calmels C Calmels C, Holmes P, Lopez E, Naman V. Chronometric comparison of actual and imaged complex movement patterns. J Mot Behav. 2006;38(5):339–48. 10.3200/JMBR.38.5.339-348 16968679

[50] Kunz BR, Creem-Regehr SH, Thompson WB. Evidence for motor simulation in imagined locomotion. J Exp Psychol Hum Percept Perform. 2009;35(5):1458–71. 10.1037/a0015786 19803649

[51] Calmels C, Fournier J. Duration of physical and mental execution of gymnastic routines. Sport Psychologist. 2001;15(2):142–150. 10.1123/tsp.15.2.142

[52] Reed CL. Chronometric comparisons of imagery to action: visualizing versus physically performing springboard dives. Mem Cognit. 2002;30(8):1169–78. 10.3758/BF03213400 12661849

[53] Minvielle-Moncla J, Ripoll H, Audiffren M. The effect of expertise on spatial and temporal representations of a choreographed dance solo. Int J Sport Exerc Psych. 2003;1(4):372–89. 10.1080/1612197X.2003.9671726

[54] Ivanenko YP, Dominici N, Daprati E, Nico D, Cappellini G, Lacquaniti F. Locomotor body scheme. Hum Mov Sci. 2011 4;30(2):341–51. 10.1016/j.humov.2010.04.001 21453667

[55] Iosa M, Zoccolillo L, Montesi M, Morelli D, Paolucci S, Fusco A. The brain's sense of walking: a study on the intertwine between locomotor imagery and internal locomotor models in healthy adults, typically developing children and children with cerebral palsy. Front Hum Neurosci. 2014;8:859 10.3389/fnhum.2014.00859 25386131 PMC4209890

[56] Williams SE, Cumming J, Ntoumanis N, Nordin-Bates SM, Ramsey R, Hall C. Further validation and development of the movement imagery questionnaire. J Sport Exerc Psychol. 2012 10;34(5):621–46. 23027231 10.1123/jsep.34.5.621

[57] Malouin F, Jackson PL, Richards CL. Towards the integration of mental practice in rehabilitation programs. A critical review. Front Hum Neurosci. 2013;7:576 10.3389/fnhum.2013.00576 24065903 PMC3776942

[58] BeauchetO, AllaliG, LaunayC, HerrmannFR, AnnweilerC. Gait variability at fast-pace walking speed: a biomarker of mild cognitive impairment? J Nutr Health Aging. 2013;17(3):235–9. 10.1007/s12603-012-0394-4 23459976

